# Supplementary material for: Reconstruction of genetically identified neurons imaged by serial-section electron microscopy
Source: eLife. 2016 Jul 7;5:e15015. doi: 10.7554/eLife.15015 (PMC4959841; doi:10.7554/eLife.15015)
Supplement: Supplementary file 1. — DOI: http://dx.doi.org/10.7554/eLife.15015.012 [file elife-15015-supp1.docx]

**Suppl. File. 1 AAV peroxidase constructs**

AAV constructs Cre dependence Peroxidase cDNA

_________________________________________________________________________________________________

AAV-CAG-DIO-APX Cre-dependent APX (Pea ascorbate peroxidase, dimeric)

AAV-CAG-DIO-APEX2NES Cre-dependent APEX2NES (Soybean ascorbate peroxidase, monomeric, NES)

AAV-CAG-DIO-erHRP Cre-dependent erHRP (Horseradish peroxidase, ER)

_________________________________________________________________________________________________

All of plant peroxidase cDNAs are codon-optimized for expression in mammalian cells. NES, nuclear export signal. APEX2 is more robust monomeric ascorbate peroxidase than APX. erHRP has a signal sequence from human immunoglobulin kappa light chain and N175S mutation to its activity in addition to the KDEL enhance endoplasmic reticulum retention signal (ER).
