# Supplementary material for: Reconstruction of genetically identified neurons imaged by serial-section electron microscopy
Source: eLife. 2016 Jul 7;5:e15015. doi: 10.7554/eLife.15015 (PMC4959841; doi:10.7554/eLife.15015)
Supplement: Supplementary file 4. — DOI: http://dx.doi.org/10.7554/eLife.15015.015 [file elife-15015-supp4.docx]

**Suppl. File. 4 Table of Content - Artemis Live Scripts.mlx**

Matlab live script that provides a walk-through of the computational pipeline

**Code/otsu.m:** Cluster-based image segmentation code.

**Code/cal.m:** Cluster-based image segmentation code.

**Code/Artemis-NETFRAMEWORK-Code.vb:** Database segment search code.

**Code/BaseReconstructor.m:** Graph-based reconstruction code.

**EM:**  Original EM images.

**Threshold:** Thresholded images preserving ARTEMIS markers.

**Otsu:** Segmented images.

**BigParts:** Images of identified large segments.

**ANM:**  images showing the progression of a reconstruction.

**GT:** Ground-truth images for comparison.

**db.csv:** example database cataloguing meta-data associated with identified segments.

**Bigparts.csv:** subset of database reflecting identified large segments.

**smp01.csv:** example output from Code/Artemis-NETFRAMEWORK-Code.vb.

***.png files:** example images for Artemis live script.
